# Supplementary material for: Discovery of an Insect Neuroactive Helix Ring Peptide from Ant Venom
Source: Toxins (Basel). 2023 Oct 5;15(10):600. doi: 10.3390/toxins15100600 (PMC10610885; doi:10.3390/toxins15100600)
Supplement: Supplementary file 1 [file toxins-15-00600-s001.zip › toxins-2642333-supplementary.pdf]

SUPPLEMENTARY INFORMATION

Discovery of an Insect-Neuroactive Helix Ring Peptide from Ant Venom

This PDF file includes:

Figure S1	**Impact of injection of U_{11} on aphid fitness**	Page 2
Figure S2	**U_{11} insecticidal activities by ingestion**	Page 3
Figure S3	**Impact of injection of U_{11} on aphid and weevil**	Page 3
Figure S4	**TOCSY and NOESY spectra of U_{11}**	Page 4
Figure S5	**Natural abundance 15N-SOFAST HMQC spectrum of U_{11}**	Page 5
Figure S6	**Natural abundance 13C HSQC spectrum of U_{11}**	Page 6
Figure S7	**Amino acid sequence alignment of U_{11} peptides**	Page 7
Table S1	**Experimental restraints and structural statistics of U_{11}**	Page 8

A.

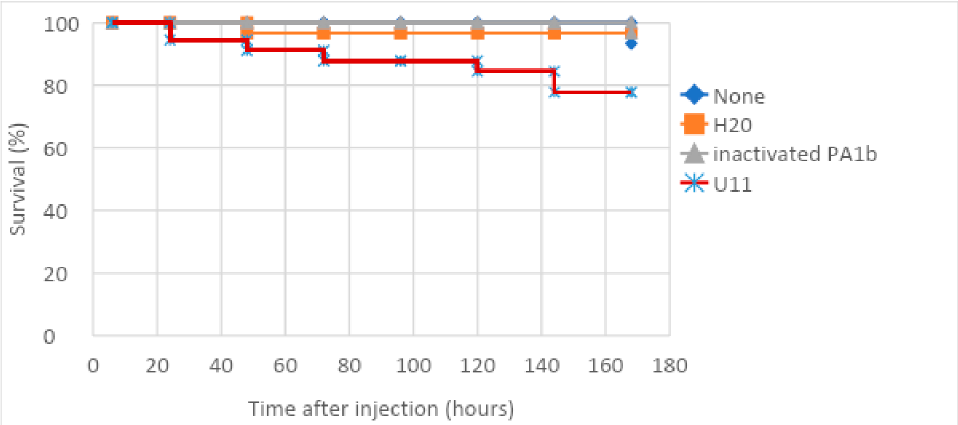

B.

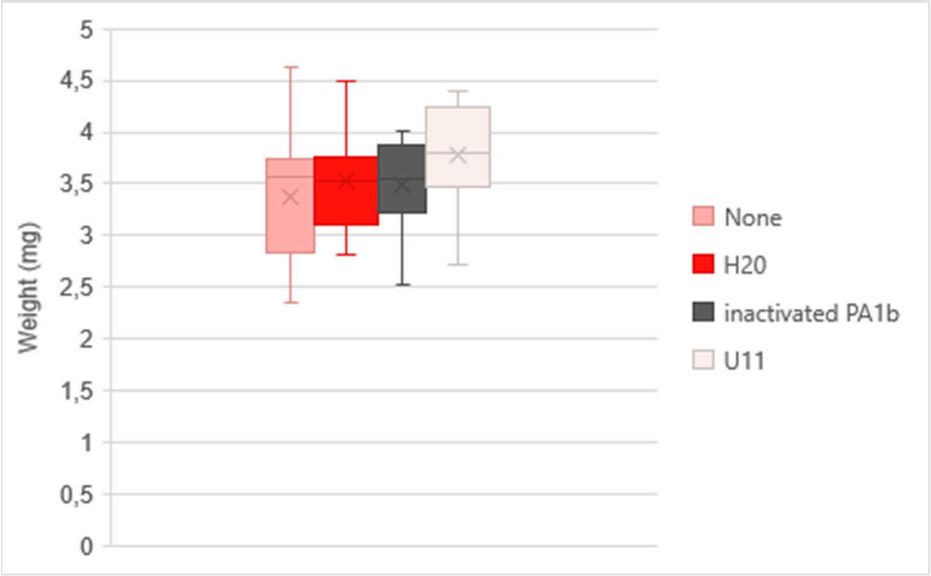

Figure S1. Impact of injection of U$_{11}$ on aphid fitness. (A) Survival curves. The different lines represent the survival curves for the different groups of aphids: (*i*) non-injected aphids, (*ii*) aphids injected with sterilized water, (*iii*) aphids injected with the negative control (PA1b inactivated peptide) and (*iv*) aphids injected with the U$_{11}$ (n = 30 aphids per condition). Aphids were monitored daily. Survival data were analyzed with the R survival and survminer packages and curves were compared using the log rank test. (B) **Aphids' weight 7 days after injection**. Results are displayed as box plots where central lines represent the medians, boxes comprise the 25–75 percentiles and whiskers denote the range; (n = 10 aphids per condition). Because the final aphid mass data did not meet the conditions for normality and homoscedasticity (Shapiro wilk and bartlett test), they were analyzed using the global nonparametric Kruskal-Wallis test, followed by a Wilcoxon multiple comparisons test with a Bonferroni correction.

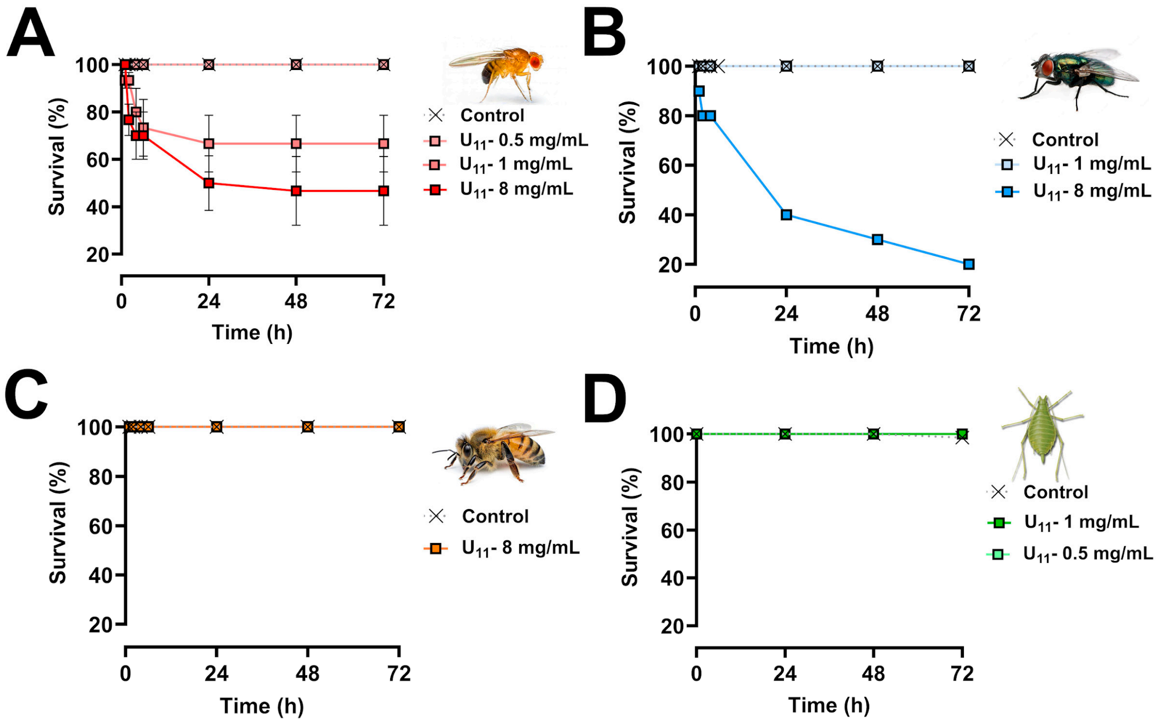

Figure S2. U_{11} insecticidal activities by ingestion. Survival rates of *D. melanogaster* **(A),** *L. caesar* **(B),** *A. mellifera* **(C)** and *A. pisum* **(D),** monitored for three days after U_{11} ingestion.

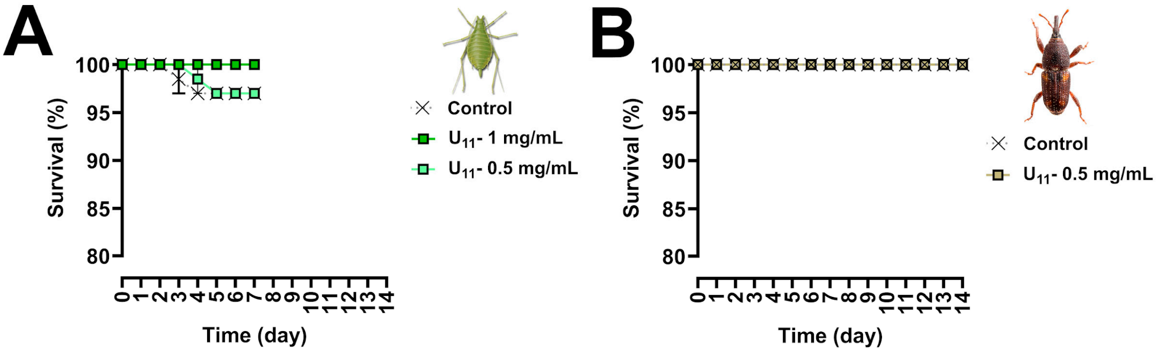

Figure S3. Impact of ingestion of U_{11} on aphids and weevils. Survival rate of aphids (*A. pisum*) and weevils (*S. orizae*) fed for seven and 14 days, respectively, with artificial diet completed with the peptide U_{11}.

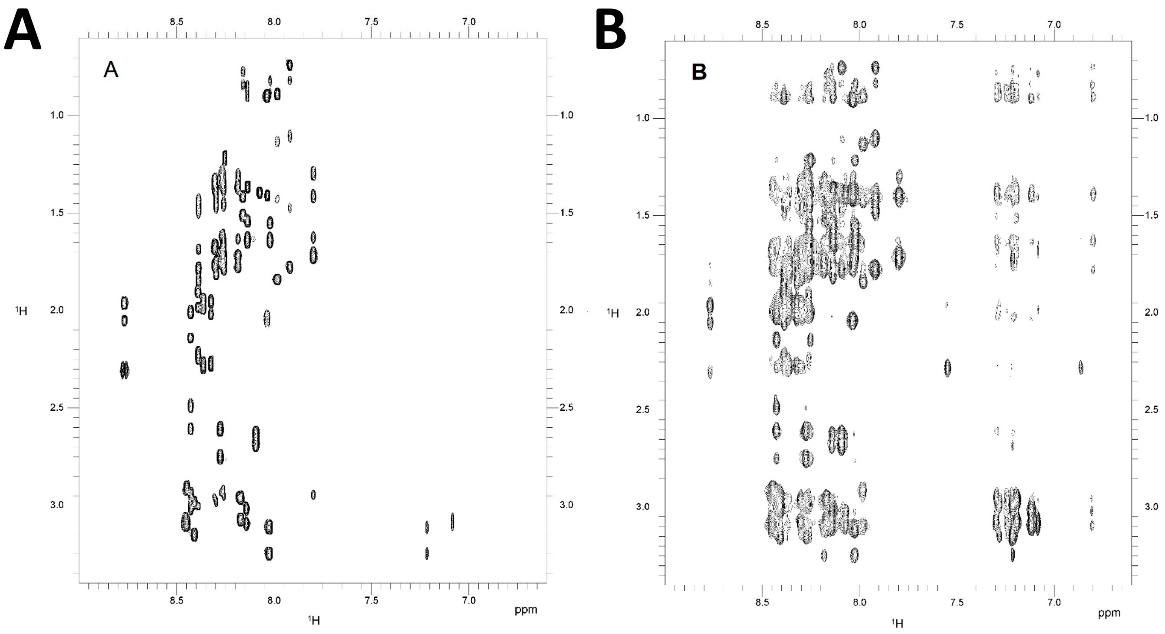

Figure S4. HN/alphatic regions of the 2D ^{1}H-NMR spectra of U_{11}. A) TOCSY spectrum and B) 100-ms mixing time NOESY spectrum of U11 in 90% H2O/10% D20 at 298 K, pH 5.1.

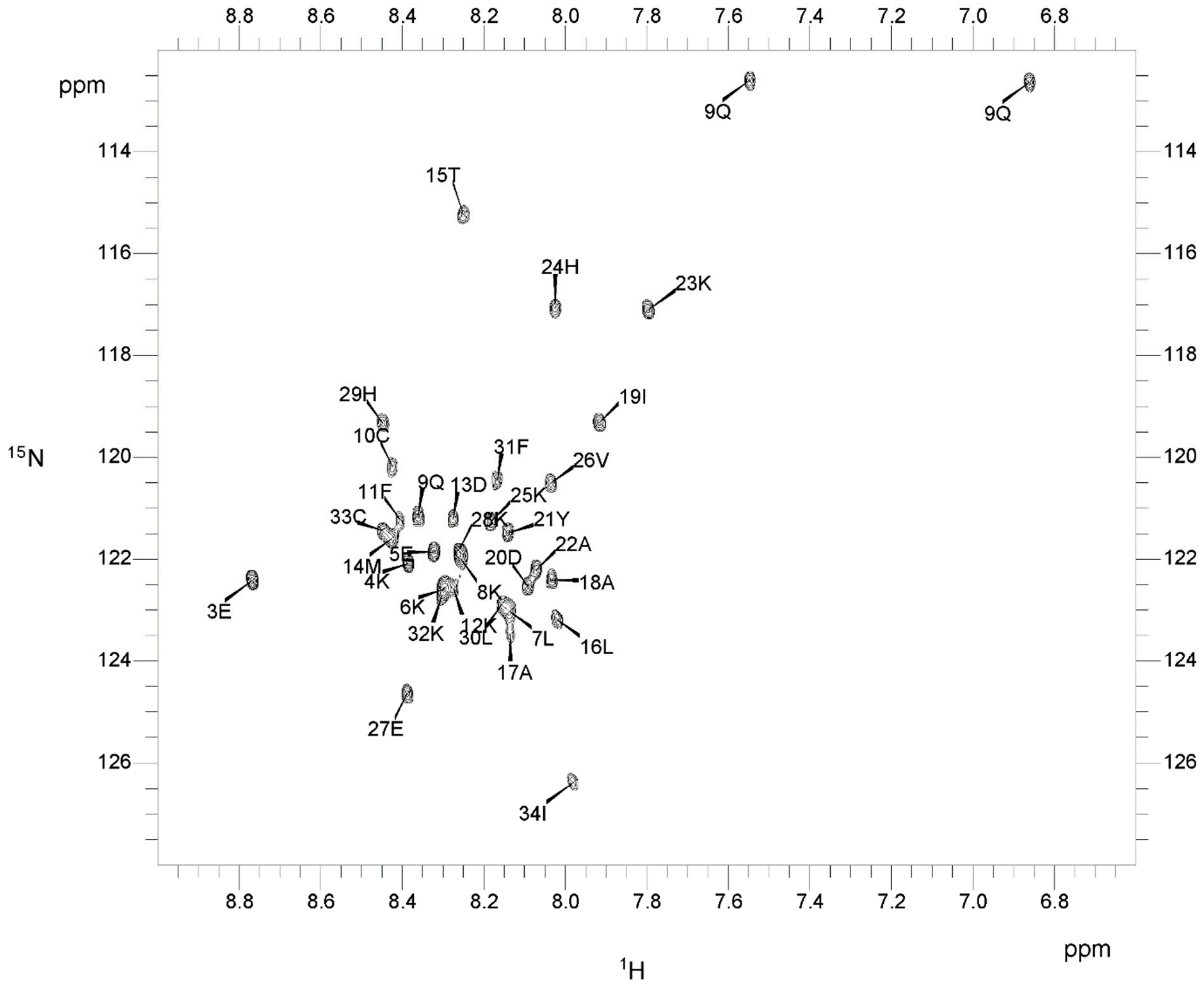

Figure S5. Natural abundance ^{15}N-SOFAST HMQC spectrum of U_{11} in 90% H2O/10% D20 at 298 K, pH 5.1. The assignment of residues to cross-peaks is indicated in one-letter code (The same region of the ^{1}H spectra is shown in the F2 dimension as in Figure S4).

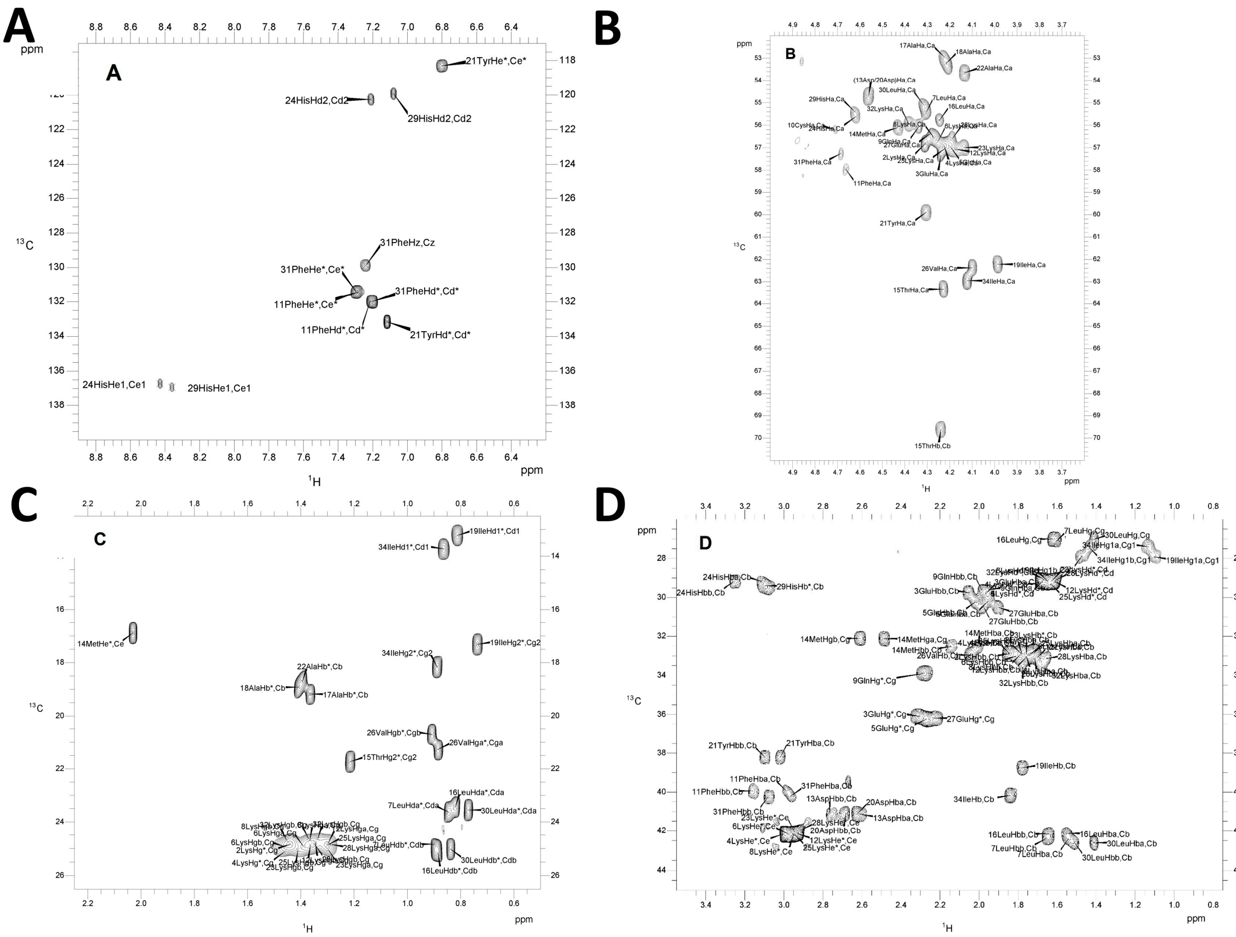

Figure S6. Natural abundance ^{13}C HSQC spectrum of U_{11} in 90% H_2O/10% D20 at 298 K, pH 5.1. A) Aromatic region. B) Hα/Cα and threonines Hβ/Cβ region, illustrating how the 13C-HSQC spectrum allows unambiguous assignment of Thr15 Hα and Hβ protons. C and D) Aliphatic side-chains Hβ/Cβ, Hγ/Cγ, Hδ/Cδ and Hε/Cε regions. Both panels illustrate Ile19 and Ile34 unambiguous side chain assignment.

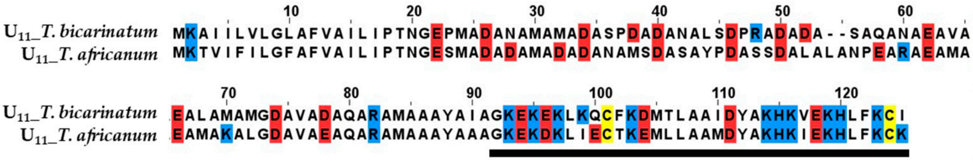

Precursor		Mature peptide	
%Id	%Si	%Id	%Si
100	100	100	100
68	76	74	85

Figure S7. Amino acid sequence alignment of U_{11} peptides. Clustalw alignment of the U_{11} precursor sequences from *Tetramorium* ant venoms. Mature peptides are underlined, lysine/arginine residues are boxed in blue, glutamate/aspartate residues are boxed in red, and cysteine residues are boxed in yellow. %Id and %Si indicate the percentage of sequence identity and sequence similarity, respectively, for the whole precursor sequence and the mature region.

Table S1. Experimental restraints and structural statistics of U_{11}. [a]: values are given as mean ± standard deviation (n = 15). [b] calculated using MOLMOL.

NMR restraints		
Distance restraints		
Total NOE	738	
Unambiguous	509	
Ambiguous	229	
Hydrogen bonds	14	
Dihedral Angle Restraints	48	
Covalent disulfide bond	Cys^{10}-Cys^{33}	
**Structural Statistics for the final 15 models of U_{11}**		
Average number of violations per structure		
NOEs ≥ 0.3 Å	0	
Hydrogen bonds ≥ 0.3 Å	0	
Dihedrals ≥ 10°; ≥ 5°	0; 2.1	
Average RMSD (pairwise, Å)[a, b]	bb (N-C_α--C′)	All heavy atoms
Whole (2-33)	0.35 ± 0.08	0.75 ± 0.14
Ring 10-33	0.27 ± 0.09	0.60 ± 0.14
Secondary structure elements (7-9; 17-24; 27-30)	0.20 ± 0.05	0.52 ± 0.11
Ramachandran Analysis		
Most favored region and allowed region	89	
Generously allowed	10.6%	
Disallowed	0.4%	
Energies ($kcal.mol^{-1}$)[a]		
Electrostatic	-1239.1 ± 28.0	
Van der Walls	-343.9 ± 4.2	
Total energy	-1007.6 ± 26.4	
Residual NOE energy	43.8 ± 5.5	
